# A ray-tracing algorithm for *ab initio* calculation of thermal load in undulator-based synchrotron beamlines

**DOI:** 10.1107/S160057752000778X

**Published:** 2020-07-14

**Authors:** Luca Rebuffi, Xianbo Shi, Manuel Sanchez del Rio, Ruben Reininger

**Affiliations:** a Argonne National Laboratory, 9700 South Cass Avenue, Lemont, IL 60439, USA; b ESRF, 71 Avenue des Martyrs, 38043 Grenoble, France

**Keywords:** thermal load, simulation, algorithm, synchrotron radiation

## Abstract

The *OASYS* suite and its powerful integration features are used to implement a ray-tracing algorithm to accurately calculate the thermal load in any component of an undulator-based synchrotron beamline. This is achieved by sampling and converting the *SRW* source of a given energy into a *Shadow* source and using the latter code to ray trace the full beamline. The accuracy of the algorithm is proved by reconstructing the full undulator radiation distribution through an aperture and comparing the result with direct calculaton of the total power using *SRW*. The algorithm is particularly suited to analyze cases with complex beamline layouts and optical elements, such as crystals, multilayers, and compound refractive lenses. Examples of its use to calculate the power load on elements of two of the feature beamlines at the Advanced Photon Source Upgrade Project and a comparison of the results with analytical calculations are presented.

## Abstract 2

The *OASYS* suite is used to implement a ray-tracing algorithm to calculate the thermal load in undulator-based synchrotron beamlines. The algorithm is particularly suited to analyze cases with complex beamline layout and optical elements. Examples of its use on two of the feature beamlines at the Advanced Photon Source Upgrade Project are shown.

## 1. Introduction   

The Advanced Photon Source Upgrade (APS-U) project encompasses the construction of a storage ring that will reduce the electron beam emittance by a factor of ∼75 as well as increasing the storage ring current by a factor of two (APS-U, 2019 ▸). The small emittance will be obtained by replacing the present 7 GeV storage ring lattice with a 6 GeV multi-bend achromat (MBA) lattice (Einfeld *et al.*, 2014 ▸). The most significant improvements are the decrease of the horizontal source size by a factor of ∼20, the increase of the coherent fraction by two orders of magnitude, and the flux increase by a factor of two. Several feature beamlines have been designed to exploit these new characteristics. One of the major concerns is the thermal stability of optical elements and radiation safety system (RSS) components, such as photon masks and beam stops. The power distribution of undulator radiation and its propagation through simple optical components can be calculated analytically with *XOP* (Sanchez del Rio & Dejus, 2011 ▸) or similar software, and by combining analytical calculations with ray tracings as in *IDPower* (Reininger, 2001 ▸). However, existing tools are less accurate when considering elements with a complex reflectivity or absorption profile (*e.g.* crystal/multilayer monochromators and compound refractive lens) or when strong diffraction effects are present. Here we approach the problem by creating an *ab initio* procedure where no assumptions must be made and the behavior of the optical elements is accurately reproduced by robust simulation tools. The *OASYS* (*ORange SYnchrotron Suite*) environment (Sanchez del Rio & Rebuffi, 2019 ▸) is chosen to integrate all elements of the procedure, as well as to collect, display, and store the results.

### 1.1. The *OASYS* integrated environment   

**Figure 1:**
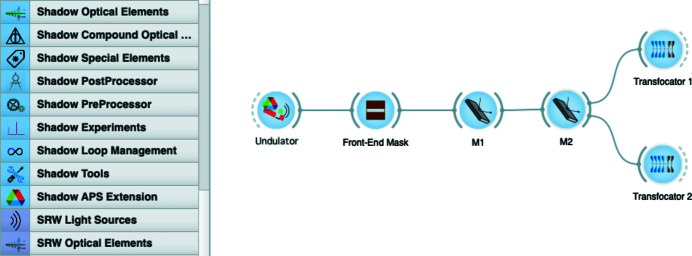
*OASYS* graphic user interface: the elements of the beamline are the active visual objects (widgets), and the photon beam transport is realized by connecting them with wires.

Since 2013, *OASYS* has been developed as a versatile, user-friendly, and open-source graphical environment for modeling X-ray experiments by optical simulations (Rebuffi & Sanchez del Rio, 2017*a* ▸). Its concept stems from the need of modern software tools to satisfy the demand of performing more and more sophisticated analysis and design of optical systems for fourth-generation synchrotron and free-electron laser (FEL) facilities. The *OASYS* workflow mechanism describes a beamline by representing sources and optical elements as active visual elements (widgets) and the photon beam as the data content passing through their connections (wires). An example of the *OASYS* user interface is shown in Fig. 1 ▸.

The ultimate purpose of *OASYS* is to integrate in a synergetic way the most powerful calculation engines available to perform virtual experiments in a synchrotron beamline, from the electron emission to the sample interaction. For X-ray optics, *OASYS* integrates diverse strategies via the implementation of different simulation tools (*e.g.* ray-tracing and wave optics packages). It provides a language to make them communicate by sending and receiving encapsulated data (Rebuffi & Sanchez del Rio, 2017*b* ▸).

*OASYS* itself is an empty container, while application program interfaces (APIs) are released as distinct groups of widgets called ‘add-ons’, which can be installed individually by users. Two tools relevant for this study are the ray-tracing program *Shadow* (Sanchez del Rio *et al.*, 2011 ▸; Rebuffi & Sanchez del Rio, 2016 ▸) and the wave optics program *SRW* (Chubar & Elleaume, 1998 ▸; Chubar *et al.*, 2002 ▸, 2013 ▸). Moreover, *OASYS* makes the availability of different programs to calculate the individual response of each optical element and the characteristics (*e.g.* emitted flux and power) of a source. This concept of toolbox is imported from *XOP* (Sanchez del Rio & Dejus, 2011 ▸), developed since 1996, which became very popular in synchrotron facilities. All applications in *XOP* have been ported to Python and integrated into *XOPPY*. *XOPPY* interfaces undulator simulation codes such as *US*, *URGENT* (Walker & Diviacco, 1992 ▸), and *SRW*. For crystals, the add-on XRayServer implements a front-end of X-ray diffraction and scattering routines available in its widely used web server (Stepanov, 2004 ▸).

*OASYS* contains a framework providing the glossary for the definition of light sources and optical components as a common layer beyond APIs. This framework separates the physical description of elements from details of the calculation algorithm. It allows users to easily benchmark calculations by using different codes for the same simulation (Rebuffi & Sanchez del Rio, 2017*b* ▸). These common definitions and data structures made *OASYS* a platform able to combine different APIs through exchanging data and results.

## 2. The simulation algorithm   

The developed procedure relies on the fact that the radiation emitted by an undulator source shows strong photon energy dependent power and power density distributions in both position and angle (Clarke, 2004 ▸). The radiation emitted by a source, as it propagates along a beamline, is not only limited by the geometrical acceptance of the beamline elements but also modified in shape and intensity by the interaction with optical elements through reflection, refraction, absorption, and diffraction. All these phenomena show a dependence on the radiation energy and/or the incident angle. This large variability and reliance on the photon energy lead to the design of an iterative approach by sampling the energy spectrum of the source radiation. The algorithm relies on the accurate simulation of the source radiation at different energies and its propagation through various optical elements. In this work, we selected *SRW* to simulate the undulator radiation and *Shadow* to simulate the transport of radiation through optics, by combining them in the *OASYS* environment.

### 2.1. Ray tracing of the undulator source   

A new undulator source widget (see Fig. 1 ▸) is created to generate *Shadow* rays based on the spatial and angular radiation distribution calculated by *SRW* at a given energy. The angular distribution is obtained by propagating the radiation of a single electron radiation to a screen at a certain distance from the source (typically at the front-end mask location) and convoluting it with the phase space of the electron beam. The obtained intensity [in photons s^−1^ mm^−2^ (0.1% bandwidth)^−1^] as a function of the transverse coordinates (*x*. *z*) is then used to sample the angular probability distribution by normalizing to an integral value of 1 and converting spatial to angular coordinates (*x*′, *z*′) using
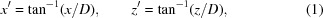
where *D* is the distance from the center of the source to the propagation screen. Since *SRW* does not create the initial wavefront inside the insertion device, obtaining the source spatial distribution requires backpropagating the wavefront to the source center and then convoluting it with the size of the electron beam.

**Figure 2:**
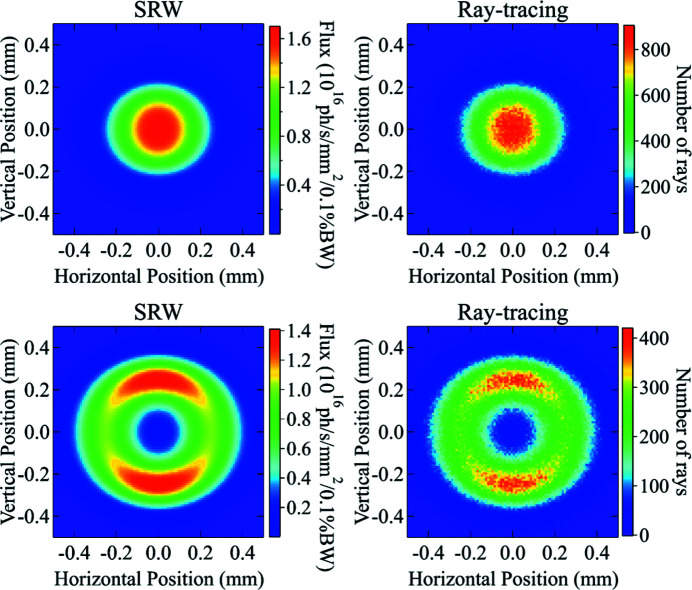
Spatial distribution of an undulator radiation on a screen at 27 m from the source, calculated with *SRW* wavefront propagation (left, top and bottom) and the ray-tracing reconstruction with *Shadow* (right, top and bottom) at the resonant energy of the first harmonic *E*_1st_ = 5 keV (top-left and bottom-left), and at the red-shifted energy *E*_rs_ = 4.95 keV (top-right and bottom-right). The undulator used in this example is U25 at APS-U described later in Table 2 ▸.

Similarly, the source spatial distribution is converted into spatial probability distribution by normalizing it to an integral value of 1. For each energy value, the two distributions obtained from *SRW* are used as probability distributions for random generators to initialize coordinates and directions of an arbitrary set of *Shadow* rays (see Fig. 2 ▸).

### 2.2. The power density calculation   

The first step of the algorithm is to calculate the spectral flux integrated on the initial screen/aperture, using the *SRW* option in *XOPPY*. The integrated spectral flux (SF) over the aperture area at photon energy *E* in units of photons s^−1^ (0.1% bandwidth)^−1^ is
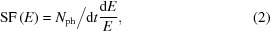
where *N*_ph_ is the number of photons, 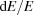 is the unit bandwidth that is commonly set to 0.1% of the energy *E*, and d*t* represents the unit time. The spectral flux (in photons s^−1^), corresponding to an energy interval [*E*, *E* + δ*E*] can be approximated with the following equation,
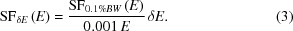
The power in the energy interval [*E*, *E* + δ*E*] is given by
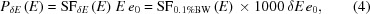
where *e*_0_ is the electric charge of an electron. The cumulated power (CP) from an initial energy value *E*_0_ up to the energy value *E*_*i*_ is
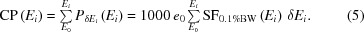
Once calculated, for any given *E*_*i*_, we can interpolate the corresponding energy step [*E*_*i*_, *E*_*i*_ + δ*E*_*i*_] that provides any arbitrary increment of power 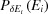. The way the cumulated power distribution is divided into power steps plays an essential role in the result of the calculation and will be discussed later. For the moment, we consider the total (cumulated) power to be divided into constant power steps, providing a corresponding series of (variable) intervals.

For every *E*_*i*_ value, the source radiation is represented by *N*_rays_ rays using the algorithm described in Section 2.1. *Shadow* computes the radiation transport along the beamline, considering all physical effects interacting with optics, such as reflection, absorption, refraction, and diffraction due to the optics size or apertures. The power emitted by the source at a given step, 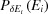, is divided by the number of rays to give the power carried by each ray. At other positions along the beamline, the power of a single ray containing effects of optical elements is given by
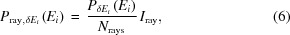
where *I*_ray_ is a number between 0 and 1 (the initial value for each ray emerging from the source) representing the intensity of the ray as modulated by *Shadow* during the ray tracing through the optical elements. Furthermore, rays can be marked as lost when they do not intercept one of the optical element or are blocked by an obstacle. Then, the power these rays carry is considered either lost or absorbed accordingly. The power density (W mm^−2^) in the energy interval [*E*_*i*_, *E*_*i*_ + δ*E*_*i*_] is given by the 2D histogram of rays in the corresponding spatial coordinates with a weighting factor equal to the power 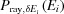 divided by the area of the binning unit (*i.e.* pixel size), or
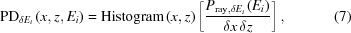
where δ*x* and δ*z* are the pixel sizes of the histogram. The total power density is obtained by iterating on the energy values *E*_*i*_ obtained from the sampling of the cumulated power spectrum and adding to the same histogram the corresponding 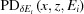 values obtained at each iteration, or
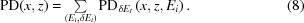
Thus, the quality of the final result depends on the sampling of the power spectrum, the number of rays for each iteration, and the sampling of the power density histogram.

#### 2.2.1. Absorbed and transmitted power   

The power and power density absorbed and transmitted by an optical element are computed by analyzing the rays before and after the optical element. The selection criteria of rays for studying different types of optics are described in Table 1 ▸.

### 2.3. Implementation of the algorithm in *OASYS*   

**Figure 3:**
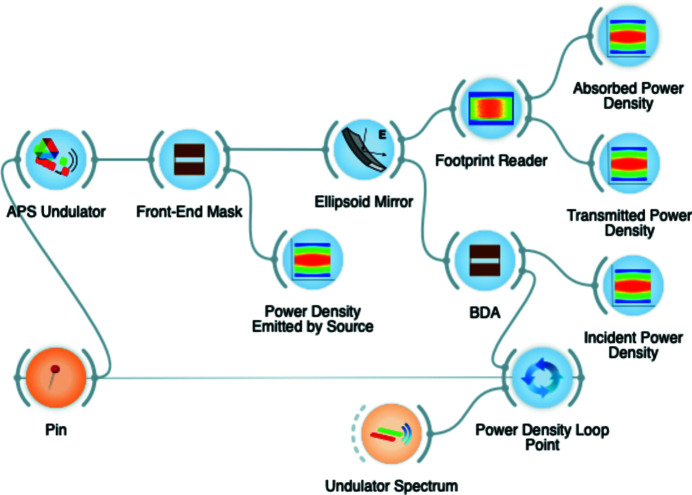
Example of the power density implementation in *OASYS*: the looping mechanism is extended by a new looping point capable of sampling the energy spectrum calculated by *XOPPY* and sending the obtained energy intervals to the new undulator widget. The undulator widget generates rays based on the spatial and angular probability distribution of the radiation calculated by *SRW*. The rays are then used to compute the power density at several locations of the beamline by using the new plotting widgets: at the front-end mask (transmitted power), on the surface of the ellipsoid mirror (transmitted and absorbed power), and at the focus where a beam defining aperture (BDA) is located (incident power).

The described algorithm is an iterative process: for every energy interval [*E*_*i*_, *E*_*i*_ + δ*E*_*i*_] in which the spectrum is sampled, the ray-tracing simulation is carried out with the results cumulated. We used the looping mechanism embedded in *OASYS* (Rebuffi & Sanchez del Rio, 2016 ▸) to produce the iteration. Several new dedicated widgets were created, including a new power density looping point to sample the energy spectrum in *N* energy intervals [*E*_*i*_, *E*_*i*_ + δ*E*_*i*_], a new undulator source widget implementing the algorithm described in Section 2.1, a plotting widget to calculate the power density from rays, and a widget dedicated to footprints calculations, which applies the criteria described in Table 1 ▸ for mirrors, gratings, and crystals. An example of the simulation layout with the new widgets is shown in Fig. 3 ▸.

#### 2.3.1. Smoothing the results   

**Figure 4:**
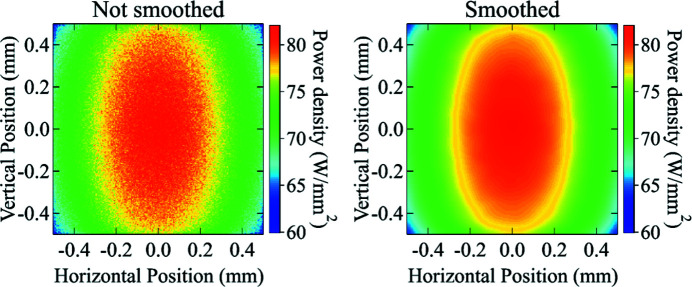
Example of smoothing with a Gaussian filter in the frequency domain to the power density distribution calculated by the cumulative ray-tracing algorithm (method scipy.ndimage.fourier_gaussian, with sigma = 4). The original histogram image is shown on the left, while the smoothed image is on the right.

The ray-tracing results are intrinsically noisy because of the user-selected finite number of random rays. Therefore, the accumulated power density distribution (histogram) has to be smoothed to remove the unphysical high-spatial-frequency noise. In our implementation, the package scipy.ndimage (Scipy, 2019 ▸), a well known and consolidated software library that provides several options to apply smoothing filters, is used in the spatial or frequency domain (Gonzalez & Woods, 2008 ▸). Fig. 4 ▸ shows an example of the smoothing process by applying a Gaussian filter in the frequency domain. The level of smoothing can be controlled by the user depending on the nature of the simulation.

#### 2.3.2. Sampling of the energy spectrum: a possible source of artifacts   

**Figure 5:**
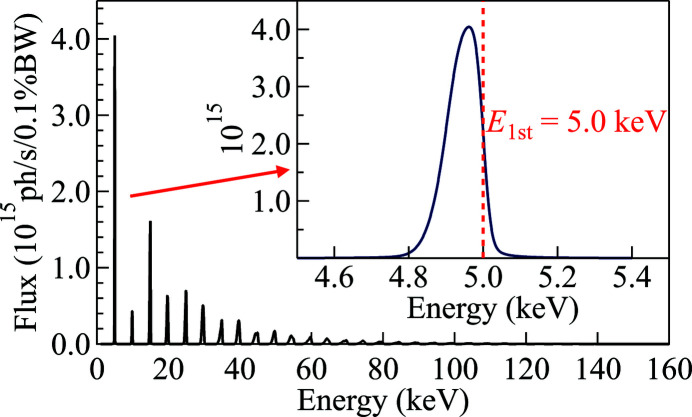
Flux spectrum of the U25 undulator source through an aperture of 1 mm × 1 mm located at 27 m from the source. The undulator *K* value is set to provide 5 keV as the resonant energy of the first harmonic. The inset shows a zoomed spectrum of the first harmonic.

For an undulator source, a critical parameter for the quality of the simulation is how the energy spectrum is sampled. For each energy interval [*E*_*i*_, *E*_*i*_ + δ*E*_*i*_] the proposed algorithm assumes that the power distribution of the whole interval can be represented by the radiation distribution at the energy *E*_*i*_. If the angular and spatial distribution is rapidly changing around the energy *E*_*i*_ and the step is too large, the algorithm can give inaccurate power distribution. This is more likely to happen around the resonance energy of the undulator harmonics, where the radiation at red-shifted energies has a ring-shape angular distribution while the radiation near the resonance energy has a near Gaussian distribution. Therefore, it is crucial to have fine enough δ*E*_*i*_ steps in the simulation, especially when dealing with the full harmonic spectrum. To verify the quality of the energy sampling, we simulated the undulator radiation through a front-end mask for a single harmonic and the whole spectrum. The parameters of the U25 undulator source, when the first harmonic is tuned at 5.0 keV, and the APS-U electron beam are summarized in Table 2 ▸. The front-end mask is positioned at 27.0 m from the source and has an opening of 1.0 mm × 1.0 mm. The calculated energy spectrum is shown in Fig. 5 ▸.

**Figure 6:**
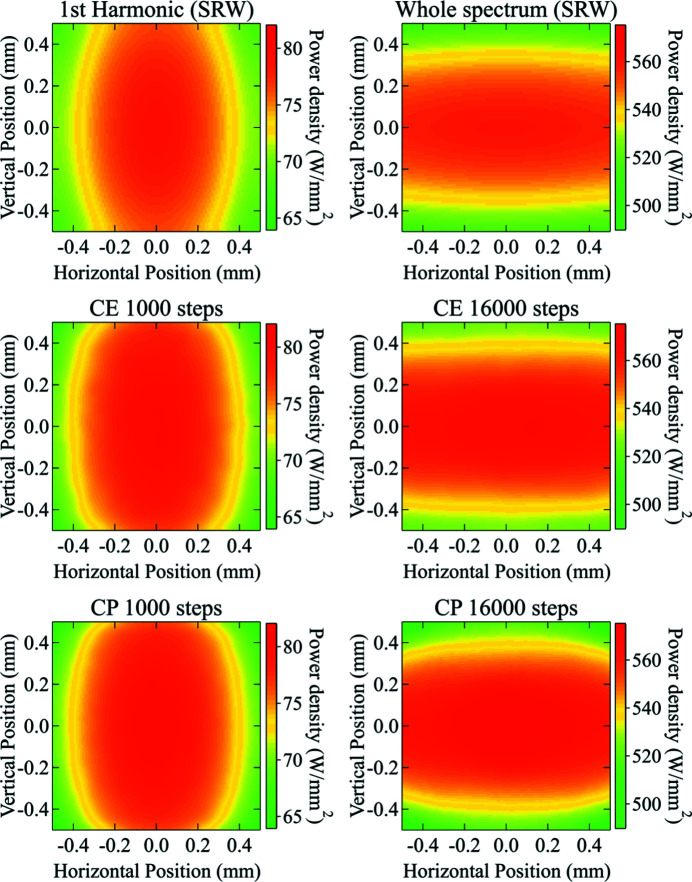
Power density distribution of the undulator radiation emitted in the energy range (4.4 < *E* < 5.4 keV) (left) and of the whole spectrum (1.0 < *E* < 161.0 keV) (right) through the front-end mask calculated using *SRW* (top), the algorithm with constant energy (CE) steps (middle) and with constant power (CP) steps (bottom). In the latter two methods, the sampling steps are 1000 and 16000 for the energies around the first harmonic and the whole spectrum cases, respectively.

Fig. 6 ▸ shows the calculated power density distributions of a range of energies around the first harmonic and of the whole spectrum using two different sampling criteria (*i.e.* constant power steps and constant energy steps) and the comparison with the *SRW* results.

With sufficient sampling steps, both sampling criteria gave excellent agreement on the total power, the average power density, and the peak power density for both the single harmonics and the whole spectrum cases (see Tables 3 ▸ and 4 ▸). It is worth noting that there is a small but still significant difference between the *SRW* calculation and our algorithm of the whole spectrum (see Table 4 ▸), that is originated by having used a portion of the spectrum corresponding to 99% of the total.

The quality of the result was studied as a function of the sampling step. In the constant energy step case, the step size needed to provide accurate results is ∼1 eV (a few thousand steps) for a single harmonic (a few keV span) and ∼10 eV (a few tens of thousand steps) for the whole spectrum (up to 150–200 keV). A similar number of steps is needed for the constant power step case as well. An undersampling will lead to artifacts, especially when the energy range is large. The artifacts are more significant for calculating the power density distribution of a beam with a large cross section, such as the footprints on the mirror surface and the divergent beam far from the source. On the other hand, the power calculation of a focused beam is less affected by a small number of steps, being the whole radiation directed in the same and typically small region of space. To provide a quality check on the simulation, the power density distribution calculated by *SRW* for every energy step is stored and plotted for checking the sufficiency of the sampling.

## 3. Synchrotron radiation beamlines use cases   

The validity of the algorithm has been demonstrated above by the simple case study. Comparing with other existing codes, the advantage of the present method is its capability of dealing with complicated layouts and optical elements and the accurate simulation of the power density on focal planes or samples. These cases include the presence of chromatic focusing optics (with energy-dependent focal length), optics with complex transmissivity profiles, and when the diffraction effects of the optics are apparent. In this section, we show the power calculation of two APS-U feature beamlines, the In Situ Nanoprobe (ISN) beamline and the X-ray photon correlation spectroscopy (XPCS) beamline, as examples.

### 3.1. ISN beamline power calculation   

**Figure 7:**
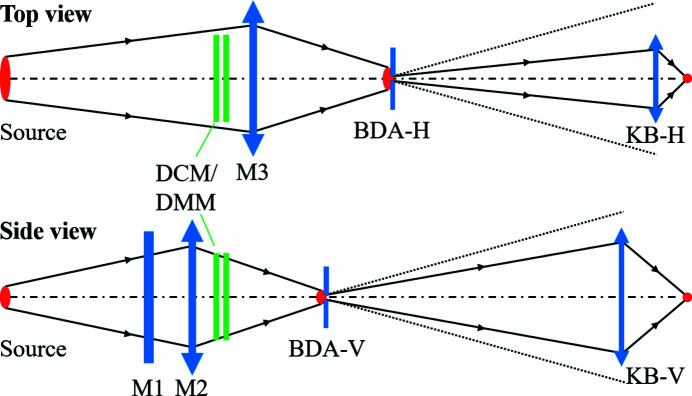
Schematic layout of the ISN beamline, showing the undulator source (on the left), the focusing mirrors (M1, M2, M3, KB-V, KB-H) and the slits (BDA-H and BDA-V).

The ISN beamline (Maser *et al.*, 2018 ▸) is designed to deliver a coherent beam to focus at the sample position with a spot size of 20 nm × 20 nm. The U25 undulator source is set to provide 17 keV photon beam at the third harmonic. Fig. 7 ▸ shows the layout of the beamline with the relevant elements listed in Table 5 ▸.

**Figure 8:**
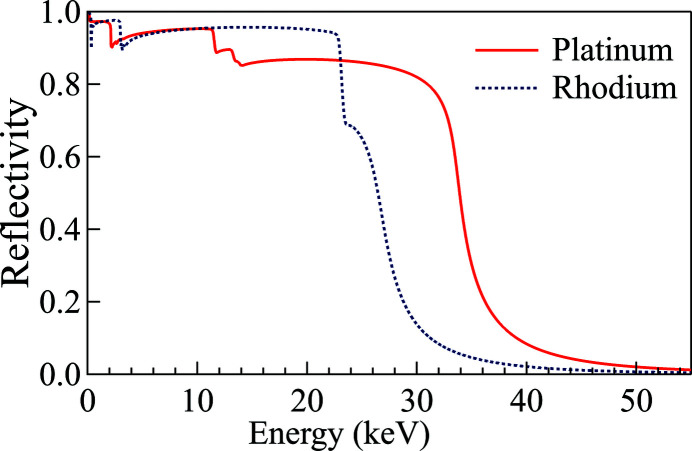
Reflectivity profiles of platinum (solid curve) and rhodium (dotted curve) coatings for a grazing angle of 2.5 mrad calculated by *XOPPY*.

The ISN beamline consists of multiple elements. M1 (high-heat-load mirror, flat) and M2 (pink-beam mirror, vertically focusing elliptical cylinder) are vertically reflecting mirrors. M3 is a horizontally reflecting, horizontally focusing elliptical cylinder. DCM and DMM are a double-crystal monochromator and a double-multilayer monochromator, respectively. BDA-V and BDA-H are the vertical and horizontal beam-defining apertures. NF-KB mirrors are the nanofocusing mirrors in the Kirkpatrick–Baez configuration (Kirkpatrick & Baez, 1948 ▸). The mirrors M1 and M2 were simulated with a rhodium coating to maximize the transmitted power at 17.0 keV (see Fig. 8 ▸). The DCM is composed of two Si(111) crystals, while the DMM consists of two multilayers with 300 Mo/B_4_C bilayers on Si substrate and a *d*-spacing of 25 Å (9 Å/16 Å). The M3 mirror was simulated with a platinum coating. The BDAs were represented as screens on which the incident power was calculated. The mirror reflectivity curves and the diffraction profiles of the two monochromators are shown in Figs. 8 ▸ and 9 ▸, respectively.

#### 3.1.1. Thermal load on optical elements with white, pink and monochromatic beams   

Power density distributions at different optics positions along the ISN beamline were calculated using the new algorithm. The absorbed power on the surface of the two mirrors (M1 and M2) and the first crystal of each monochromator is shown in Fig. 10 ▸. The incident power on the BDA-V and BDA-H were simulated with the two different monochromators (DCM and DMM) and shown in Fig. 11 ▸. Detailed discussions on the shape of these power distribution profiles will be explained in later sections.

The total power on the BDAs, which scales with the energy bandwidth of the monochromator, is near two orders of magnitude higher with the DMM. Also, the size of the power distribution profile is significantly different comparing the DMM and DCM cases (see Fig. 11 ▸). This difference is visible because the *ab initio* algorithm takes into account the correlation of the properties of the optics with both the energy and the angle of incidence of the radiation. A summary of the obtained beam sizes and peak power densities is shown in Table 6 ▸.

#### 3.1.2. Thermal load on mirrors and monochromators: contributions from single harmonics   

This section shows the importance of *ab initio* calculations for the fourth-generation synchrotron sources, which have complex radiation profiles even within a small acceptance aperture. The algorithm allows to interpret the complex power density absorbed by the optical elements by studying the contribution of the different harmonics.

The absorbed power on the first DCM crystal and the first DMM multilayer from individual harmonics was simulated and shown in Figs. 12 ▸ and 13 ▸, respectively. Since the M1 and M2 mirrors reject high photon energies, only the first four harmonics of the undulator radiation have a significant contribution on the first element of each monochromator. The results are summarized in Table 7 ▸.

**Figure 9:**
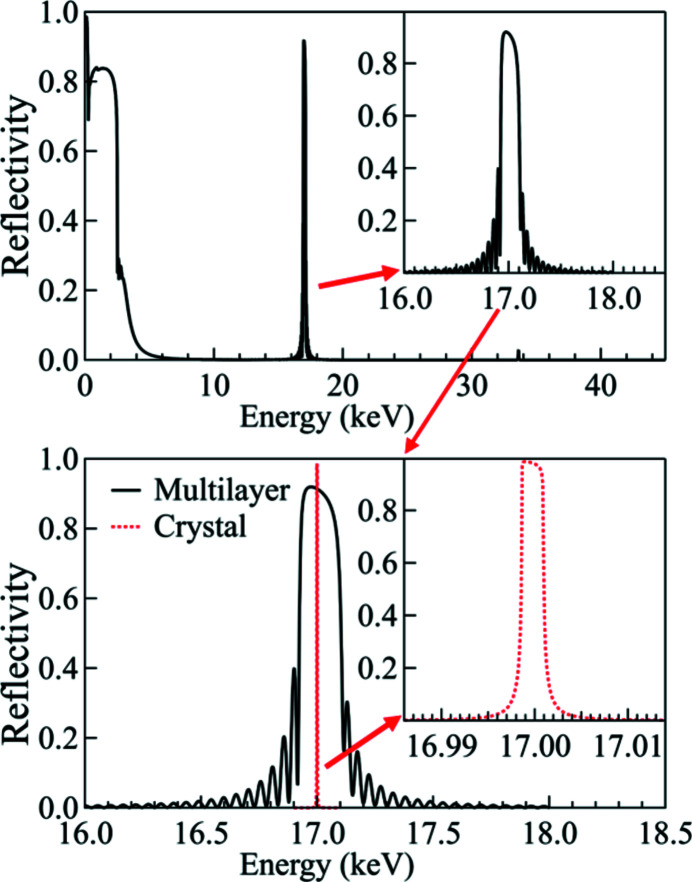
Reflectivity profile of the Mo/B_4_C multilayer (solid curve, top): the inset shows detail around the central energy (*E*_3rd_ = 17 keV). Diffraction profile of the Si(111) Bragg crystal (dotted curve) compared with the reflectivity of Mo/B_4_C multilayer (bottom): the inset shows detail around the central energy.

**Figure 10:**
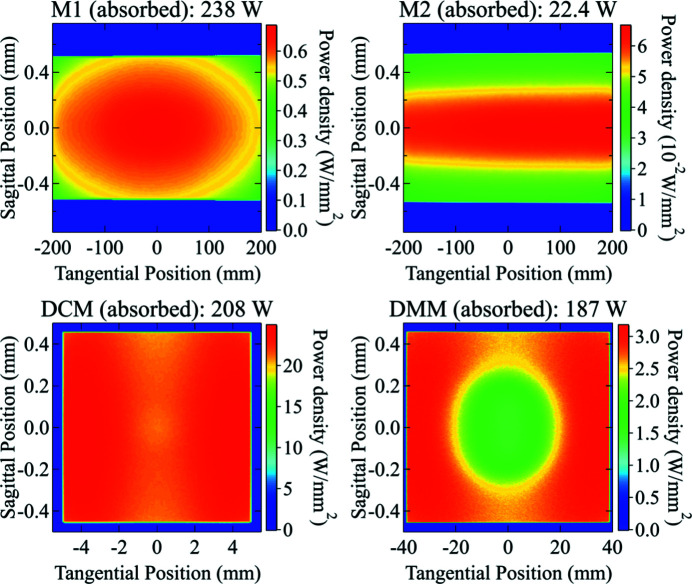
Calculated absorbed power density distribution on the surface (footprint) of M1 (top-left), M2 (top-right), the first crystal of DCM (bottom-left), and the first crystal of DMM (bottom-right). The total absorbed power is 238 W and 22.4 W, 208 W, and 187 W, respectively.

On both monochromators, the first harmonic delivers an almost uniform power distribution, while the second and fourth harmonics are responsible for the higher absorbed power near the left and right edges of the surface (see Fig. 10 ▸), because of their divergence distribution. It is worth noting that the absorbed powers on the two monochromators show very similar values, as expected for near total absorption of the radiation. However, the multilayer has a small residual mirror-like reflectivity around the first-harmonic energy (5.67 keV) (see Fig. 9 ▸), which is responsible for the lower total absorbed power and the different shape of the power density distribution. The third harmonic, which has a narrow distribution around the optical axis, forms a central ‘pit’ in the absorbed power distribution. Since the multilayer diffracts a larger bandwidth, it gives a higher reflected power and thus a lower absorbed power in the central area.

Another example of this is the power density absorbed on M2 as shown in Fig. 10 ▸. The power density absorbed on M2 is higher on the positive value side of the footprint (downstream end of the mirror), which is counter-intuitive. Normally, one would expect a higher power density on the upstream end because of its slightly shorter distance to the source. This phenomenon can only be observed and explained thanks to the *ab initio* algorithm. The reflectivity of M1 and M2 shows that they transmit most of the insertion device power up to 22.3 keV. M1 absorbs nearly all of the power above 35 keV, but only part of the energy emitted by the fifth harmonic (28.3 keV), where the reflectivity varies very rapidly as a function of the incident angle. The grazing-incident angle on M1 varies from 2.52 mrad at the upstream end to 2.42 mrad at the downstream end of the mirror due to the vertical beam divergence. This is enough to generate the asymmetry on the power transmitted by M1 and consequently absorbed by M2 around the fifth harmonic of the undulator, as shown in Fig. 14 ▸.

#### 3.1.3. Thermal load on BDAs: comparison with analytical calculations   

**Figure 11:**
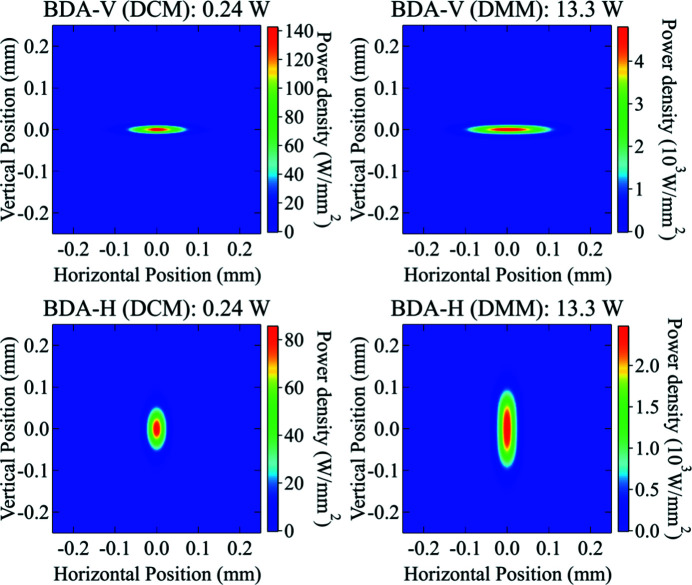
Simulated power density distribution incident on the BDA-V with DCM (top-left) and DMM (top-right) and on the BDA-H with DCM (bottom-left) and DMM (bottom-right). The total incident power on both BDAs with DCM and DMM is 0.24 W and 13.3 W, respectively.

**Figure 12:**
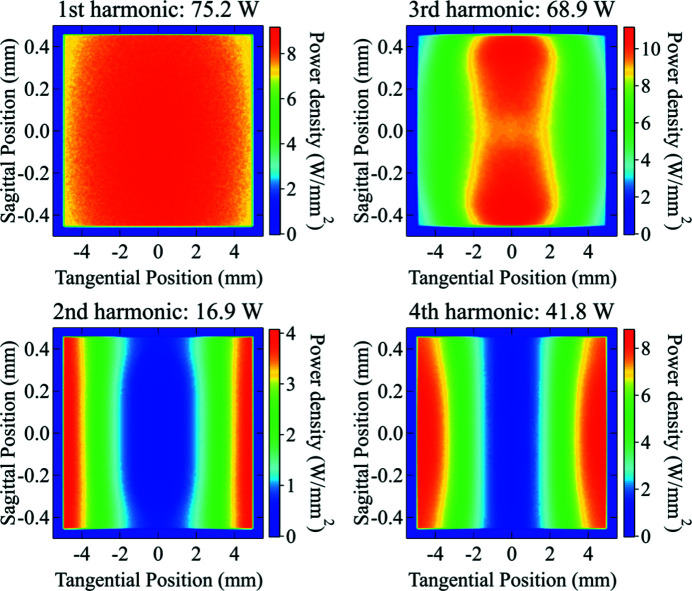
Absorbed power density distribution on the surface (footprint) of the first crystal of DCM from individual undulator harmonics. The first harmonic shows a near-uniform power distribution (top-left). The third harmonic shows a ‘pit’ in the middle because of the Bragg diffraction (top-right). Even (second and fourth) harmonics show high absorbed power near the left and right edges of the surface.

**Figure 14:**
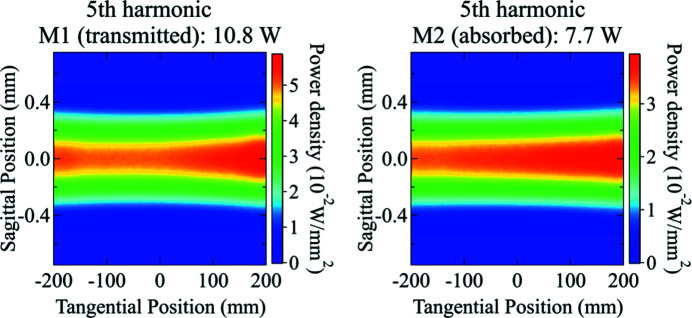
Transmitted power density distribution from the surface of the M1 mirror (left) and absorbed power density distribution on the surface of the M2 mirror (right) near the fifth harmonics of the undulator. The incident angle on M1 varies enough to generate the asymmetry on the power transmitted by M1 and absorbed by M2.

As seen above, the power and power distribution absorbed by the BDAs is significantly different when the radiation is monochromated with the DCM or the DMM. One can estimate the power transmitted by the monochromators recalling that the total power emitted by the undulator through the white beam slit is obtained from the spectrum [see Fig. 5 ▸ and equation (5)] as
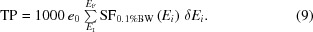
The total incident power on the BDAs can be obtained by multiplying each spectral flux value at *E*_*i*_ by the energy-dependent reflectivities of all elements, including the mirror reflectivities (see Fig. 8 ▸) and the monochromator reflectivities (see Fig. 9 ▸), or
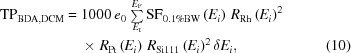


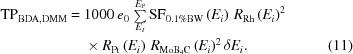
The power calculations can be simplified since one needs to consider a limited portion of the spectrum encompassing the energy where the monochromators are tuned. Namely, [16950, 17050] eV, with 0.1 eV energy step, for the DCM, and [15000, 19000] eV for the DMM, with 1 eV of energy step. The total incident power obtained analytically is 0.24 W and 12.9 W for the DCM and DMM cases, respectively. These values are in very good agreement with the *ab initio* calculation results (see Fig. 11 ▸).

Due to the narrow bandwidth of the DCM, the beam size and divergence at a resonant harmonic of the undulator can be estimated assuming Gaussian distributions (Onuki & Elleaume, 2003 ▸). For 17 keV one obtains the following:

Single-electron photon source size:
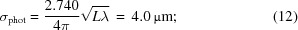
Single-electron photon source divergence:
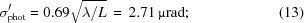
Total photon source size (*h*/*v*):
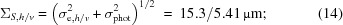
Total photon source divergence (*h*/*v*):
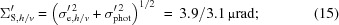
where σ_e, *h*/*v*_ and 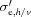 are the electron source size and divergence, respectively. Note that equations (12) and (13) are approximated to represent the nature of single-electron undulator radiation, which is fully coherent but not a perfect Gaussian beam. The emittance of the photon beam (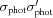) is close to 1.89λ/4π. This does not violate the inequality for any beam emittance that 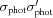 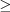 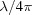, where the equals sign is satisfied for a Gaussian beam. To represent the (astigmatic) focusing optical system and compute the lateral sizes of the beam at the two BDA positions we use two ideal and orthogonal lenses. The source-to-lens (*p*) and lens-to-focus (*q*) distances for the horizontal and vertical directions are given by *p*_h_ = 35 m, *q*_h_ = 29 m, *p*_v_ = 29 m, and *q*_v_ = 26 m, respectively.

**Figure 15:**
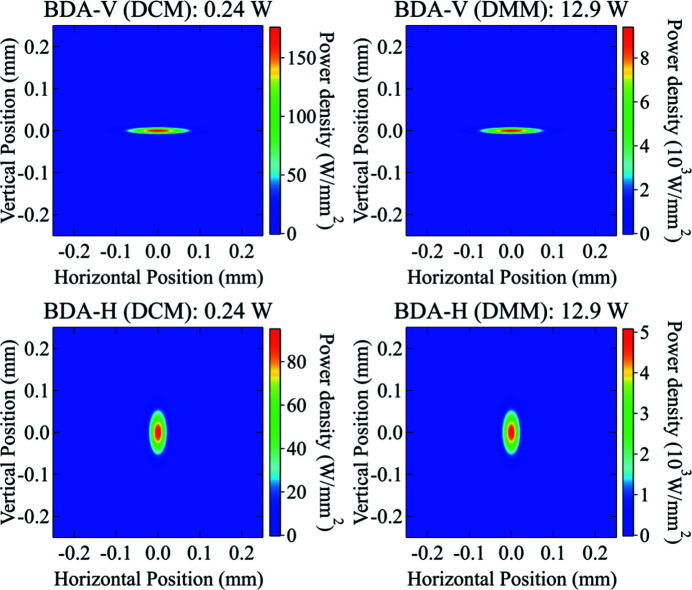
Analytically calculated power density distribution incident on the BDA-V with DCM (top-left) and DMM (top-right) and on the BDA-H with DCM (bottom-left) and DMM (bottom-right). The total incident power on both BDAs using DCM and DMM is 0.24 W and 12.9 W, respectively.

The power density distribution can be represented as a 2D Gaussian function with the total area equaling the total power TP_Transmitted_, or
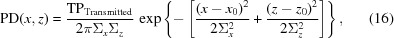
where Σ_*x*_ and Σ_*z*_ are the sigma beam sizes in the horizontal and vertical directions, respectively. The beam sizes at different locations of the beamline can be obtained analytically under geometric optics approximation by the following:

Σ at the focus:
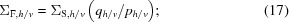
Σ′ after the lens:
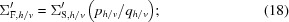
Distance between foci:
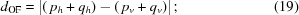
Σ out of focus:
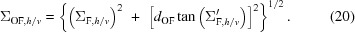
The calculated sizes and peak power density at the BDA positions for the DCM are shown in Table 8 ▸; the power density distributions are shown in Fig. 15 ▸. We have also included in the table and figure the corresponding values for the DMM.

The calculated values for the beam sizes and power densities in the DCM case are in fair agreement with the *ab initio* calculations. Clearly, the values obtained for the DMM case are far from the *ab initio* results since the beam transmitted by this monochromator has a larger bandwidth, and therefore larger size and divergence than those given in equations (17)–(20). The error in the power densities incident on the BDAs is higher by near a factor of two using the analytical equations.

#### 3.1.4. Thermal load at the sample   

The accurate simulation of the radiation power on the sample and endstation optics is essential for the instrumentation design and experimental preparation. The nanofocusing KB mirrors are designed to collect the coherent fraction of the photon beam and to provide diffraction-limited focusing. In this situation, the spatial distribution of the radiation at the sample (focus) position cannot be calculated by *ShadowOui* with a pure ray tracing, but the diffraction correction provided by the Hybrid method is necessary (Shi *et al.*, 2014 ▸; Rebuffi & Sanchez del Rio, 2016 ▸).

The power density was simulated at two locations using the DMM as the monochromator: on the beryllium window at the entrance of the sample chamber and at the sample position (see Table 5 ▸). The entrance window needs to be able to sustain the power loading and preserve the wavefront and coherence of the converging beam. The knowledge of the power at the sample position is essential for the experimental design and data collection.

**Figure 16:**
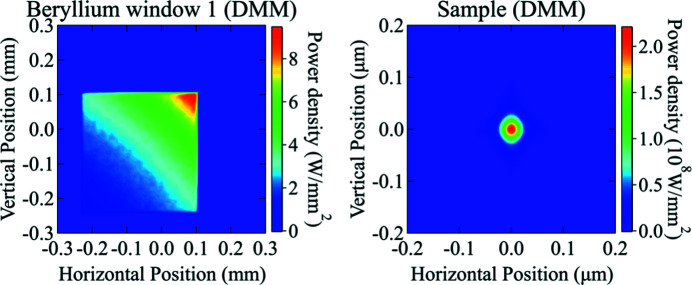
Simulated power density distribution incident on the beryllium window at the entrance of the sample chamber (left) and on the sample (right). The total incident power is 0.37 W.

Fig. 16 ▸ shows the simulated power density distribution at the two chosen locations with a total power of 0.37 W emerging from the KB mirrors. The power distribution on the beryllium window shows a typical out-of-focus beam shape downstream of nano-focusing KB mirrors. In this case, both the power and power density absorbed by the window is low. On the other hand, the power density impinged on the sample has a peak value of ∼220 MW mm^−2^, which may become a limiting factor for the sample selections. These simulations provide inputs for the necessary finite-element analysis (FEA) to determine the feasibility of the heat load management scheme. It is worth noting that diffraction effects are correctly taken into account and visible in the focal spot, which is vital for accurate simulations.

### 3.2. XPCS beamline power calculation: pink beam focused by a transfocator   

A second example is given here on a beamline containing chromatic focusing elements, namely transfocators that contain a series of compound refractive lenses (CRLs). In the case of a transfocator focusing a pink beam, an accurate analytical calculation is very complicated since the focal distance *f* of the lens stack depends on the real part of the refractive index of the lens material (Snigirev *et al.*, 1998 ▸), or
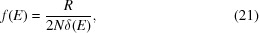
where *R* is the apex radius of the lens, and *N* is the number of lenses. The case studied here is the accidental focusing of the pink beam onto downstream elements at the APS-U XPCS beamline (APS-U, 2019 ▸).

The XPCS beamline simulations use the U21 undulator source with several *K* values providing the first harmonic energy at 10, 11, and 12 keV. The characteristics of the source are summarized in Table 9 ▸. Table 10 ▸ lists the relevant elements of the XPCS beamline.

#### 3.2.1. Thermal load on the photon shutter: pink beam focused by the transfocator   

This example simulates the accidental focusing of the pink beam (after reflection from M1 and M2 mirrors) by the transfocator on the first downstream photon shutter (PS). Three different cases with the first-harmonic energy tuned to 10, 11, and 12 keV were compared to find the maximum thermal load on the shutter. The transfocator configurations for focusing the photon beam at the PS for the three energies and the required lens specifications are listed Tables 11 ▸ and 12 ▸, respectively. *ShadowOui* assembles the transfocator as a succession of refractive interfaces (Rebuffi & Sanchez del Rio, 2016 ▸) and computes the absorption according to the optical path inside each lens. Since the transfocator can only create discrete focal distances, we chose a setup to give the closest focus at the PS location. The mirrors M1 and M2 were simulated with platinum coating.

**Figure 17:**
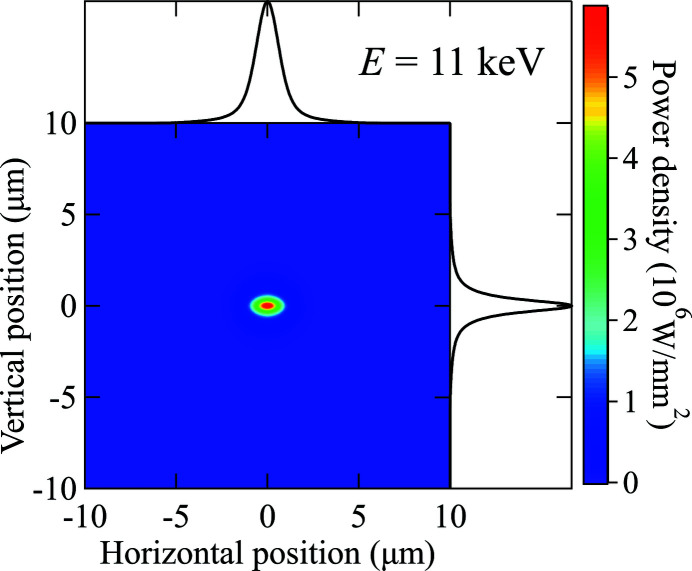
Power density distribution on the PS with the first harmonic of the undulator at *E*_1st_ = 11 keV, and the horizontal and vertical profiles.

Fig. 17 ▸ shows the simulated power density distribution on the PS for the case of *E*_1st_ = 11 keV. The integrated power density profiles in both transverse directions show a Lorentzian shape. The beam size is thus extracted as the FWHM value from a pseudo-Voigt fitting. The Lorentzian shape of the profile is caused by the chromatic aberration of the transfocator. The comparison of the three energy cases is summarized in Table 13 ▸. The 11 keV case gives the highest peak power density owing to the balance between the undulator power (higher at lower energy) and the lens transmission (higher at higher energy). Again, all these effects can be correctly accounted for by the *ab initio* algorithm.

#### 3.2.2. Thermal load on the photon shutter: comparison with analytical calculations   

The power density of the focused pink beam can be estimated analytically with the following procedures. It is worth noting that only the first-harmonic power will be properly focused near the PS, because of the chromatic focusing feature of the lenses.

(i) *Calculation of the total power transmitted through the lenses.* The total incident power through a circular aperture of the same diameter as the lens is computed by using *XOP* and multiplied by the reflectivity of the two mirrors with a platinum coating (reflectivity curves in Fig. 8 ▸).

**Figure 18:**
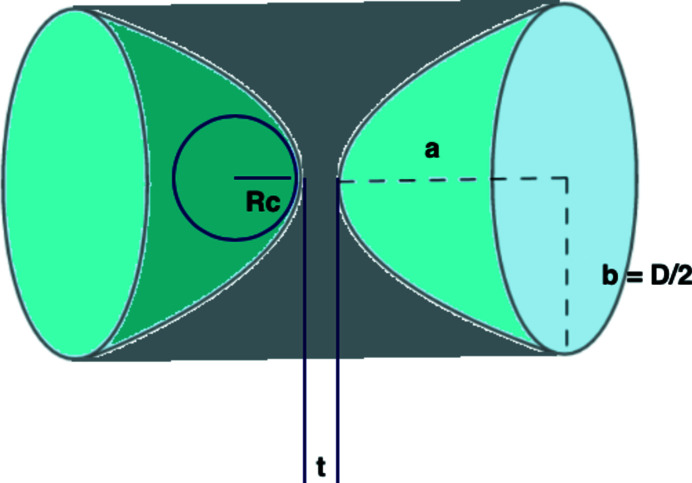
Schematic of a parabolic lens with an apex radius of *R*_c_, diameter of *D*, depth of *a*, and a minimum thickness of *t*.

The absorption of the lenses can be calculated through a mathematical integration procedure (Shi *et al.*, 2017 ▸). In this work, a simplified analytical approach is used by evaluating the effective thickness of the parabolic lens (see Fig. 18 ▸). The effective thickness is defined as the height of a cylinder which has the same volume and base area as the lens.

Using the parabolic equation 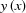 = 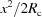, the lens depth of a single surface is 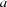 = 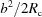 = 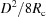. The total thickness of the lens (the height of the circumscribed cylinder) is 2*a* + *t*. Since the volume of a paraboloid is always half of the circumscribed cylinder, the volume of the remaining material is given by π*b* ^2^(*a* + *t*). The effective thickness *t*_eff_ of a single lens is thus
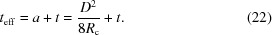
For example, a single Be lens with *R*_c_ = 100 µm, *D* = 632 µm, and *t* = 30 µm, has an effective thickness of *t*_eff_ = 529 µm, on a total thickness of 1029 µm

**Figure 19:**
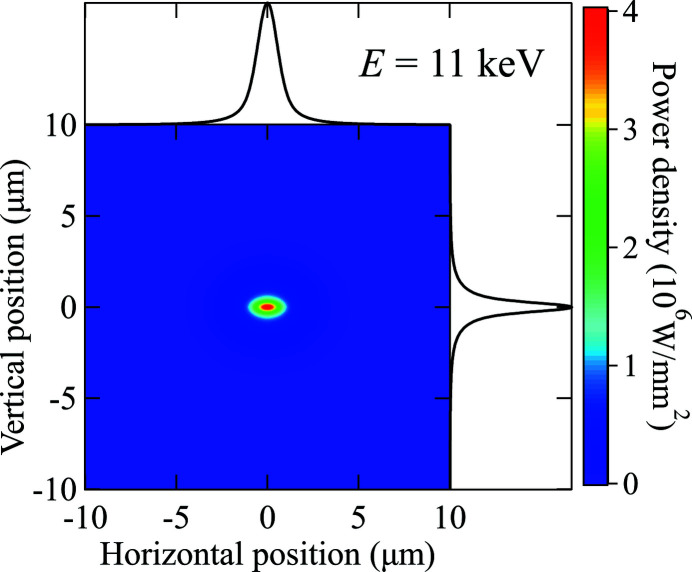
Analytical calculation of the power density distribution with first harmonic of the undulator at *E*_1st_ = 11 keV and the horizontal and vertical profiles.

The transmittance of the transfocator is then calculated by summing up contributions of all lenses as
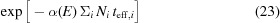
where α(*E*) = σ_tot_(*E*)ρ_Be_ is the linear absorption coefficient, σ_tot_(*E*) is the total absorption cross-section, ρ_Be_ is the beryllium density, and *N*_*i*_ is the number of lenses with the same radius *R*_c,*i*_.

(ii) *Calculation of the beam size at the PS position.* We assume that the photon beam at the resonant energy *E*_1st_ is focused at the PS location with a focal distance *q*. If the source-to-lens distance *p* is much larger than *q*, *q* can be approximated as the focal length *f*. For an energy *E*_*i*_ close to *E*_1st_, the focal position will be slightly off from *q*. From equation (21) we have
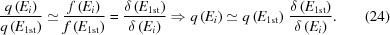
The focal spot size of each energy step [*E*_*i*_, *E*_*i*_ + δ*E*_*i*_] is calculated by using equations (12)–(20) with the focal distance *q* scaled by equation (24). The total beam size is then a sum of 2D Gaussian distributions of all energy steps. The calculation took into consideration only the portion of the spectrum corresponding to the first harmonic, since the *ab initio* procedure showed that it contributes for ∼99% of the power density distribution shown in Fig. 17 ▸. Fig. 19 ▸ shows the analytically constructed power density distribution on the PS for the case of *E*_1st_ = 11 keV (energy range: 10000–12000 eV; energy step: 1 eV). The beam profile shows the same Lorentzian shape as the one simulated with ray tracing (see Fig. 17 ▸). The results are summarized in Table 14 ▸.

The results listed in Tables 13 ▸ and 14 ▸ show a fair agreement between the analytical calculations and the *ab initio* simulation. The analytical approach can provide a fairly close total power and beam size but tends to underestimate the power density, because of longer tails on both the vertical and horizontal profiles of the power distribution. It is, therefore, suggested to use the more efficient analytical calculation to provide general guidance and to identify the worst case. The *ab initio* simulation is necessary to provide accurate power distribution for the thermal analysis and cooling design.

### 3.3. Conclusions   

The construction of fourth-generation synchrotron facilities brings many engineering challenges in beamline design to preserve the high brightness of the source. Among these challenges, the understanding of thermal load effects on optics and sample is crucial. The low emittance of these new sources implies a high power density on all the optical and safety elements, especially for the focused beam. In this work, a new tool based on an *ab initio* algorithm is introduced to simulate the power density distribution along the beamline with any source spectrum, optics element, and geometric layout.

The new tool uses the *OASYS* environment to integrate *SRW* for the source radiation simulation, *ShadowOui* for the beam propagation through beamline elements, and other tools for providing material and optical properties. It can calculate the incident, absorbed, and transmitted power density distribution at any point of the beamline, from the source to the sample. The tool takes full advantage of these software to accurately calculate the power propagation along the beamline, taking into account the physical behavior of optical elements. The validation and accuracy of the program were demonstrated by comparing the reconstructed power density distribution of the emission of an insertion device through a front-end mask with the reference results from *SRW*.

Two examples taken for our design of the APS-U beamlines illustrated the capabilities made available by the new tool. The ISN beamline features multiple optical elements, secondary focusing geometry, and diffraction-limited coherence focusing. Using the new tool, we were able to analyze the incident and absorbed power density distribution at critical points along the beamline up to the sample position. In the second case, we studied the accidental focusing of the pink beam on the radiation safety component. This *ab initio* algorithm is particularly suitable for calculating complicated optics (*e.g.* compound refractive lenses) under broad bandwidth radiation and even pink or white beam. Furthermore, the new algorithm can provide more accurate and detailed results which allow the study of extreme cases otherwise could not be calculated.

Finally, the algorithm is fully integrated into the *OASYS* environment with graphic interfaces that are easy to configure and use. The aim of the new tool is to help the thermal analysis of optical and safety components at next-generation synchrotron facilities. It will be beneficial for the many members of the *OASYS* users’ community, that often offer feedback to improve the software and to understand the most critical needs and trends on optics simulation tools for synchrotrons. All the files with the *OASYS* workspaces used in the examples of this work are available in the following public repository: https://github.com/lucarebuffi/Paper_JSR_gy5009.

## Figures and Tables

**Figure 13:**
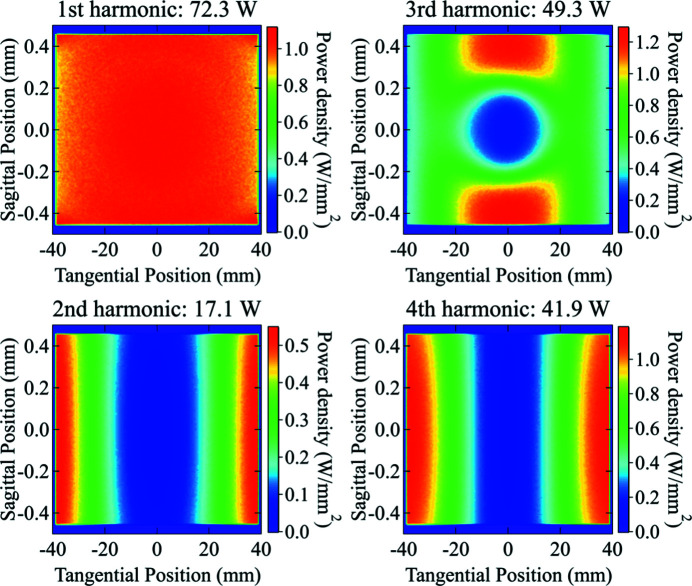
Absorbed power density distribution on the surface (footprint) of the first multilayer of the DMM from individual undulator harmonics. The first harmonic shows a near-uniform power distribution (top-left). The third harmonic has a hole in the middle because of the multilayer diffraction (top-right). Even (second and fourth) harmonics show high absorbed power near the left and right edges of the surface.

**Table 1:** Rays selection criteria to compute the absorbed and transmitted power by an optical element

Element type	Power type	Selection criteria of the rays and coordinates
Aperture (slit)	Transmitted	Good rays after the element. Coordinates in the aperture plane.
	Absorbed	Lost rays that were good before the element. Coordinates in the aperture plane.
Obstruction	Transmitted	Good rays after the element. Coordinates in the aperture plane.
	Absorbed (opaque)	Lost rays that were good before the element. Coordinates in the obstruction plane.
	Absorbed (transparent)	Good rays after the element. Coordinates in the obstruction plane. The absorbed intensity of each ray is calculated by subtracting the transmitted intensity from the intensity it had before the element.
Mirror/grating/crystal	Transmitted	Good rays after the element. Coordinates in the footprint plane on the optical surface.
	Absorbed	Good rays after the element. Coordinates in the footprint plane on the optical surface. The absorbed intensity of each ray is calculated by subtracting the transmitted intensity from the intensity it had before the element.
CRLs	Transmitted	Good rays after the element. Coordinates on a screen after the last lens.
	Absorbed	Good rays after the element. Coordinates on a screen before the first lens. The absorbed intensity of each ray is calculated by subtracting the transmitted intensity from the intensity it had before the element.

**Table 2:** Characteristics of undulator U25 at APS-U

Period (mm)	Number of periods	*K*	*E*_1st_ (keV)	Electron beam					
				*E* (GeV)	*I* (mA)	*σ*_*x*_ (µm)	σ_*y*_ (µm)	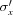 (µrad)	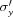 (µrad)
25	184	1.863	5	6	200	14.8	3.7	2.8	1.5

**Table 3:** Power calculation results for a single harmonic with the energy range 4.4–5.4 keV

Calculation type	Number of steps	Total power (W)	Peak power density (W mm^−2^)
*SRW*		75.9	79.1
Constant energy	1000	75.5	80.5
Constant power	1000	75.5	81.2

**Table 4:** Power calculation results for the whole spectrum with the energy range 1.0–161.0 keV

Calculation type	Number of steps	Total power (W)	Peak power density (W mm^−2^)
*SRW*		555	561
Constant energy	16000	551	569
Constant power	16000	550	575

**Table 5:** Optical components of the ISN beamline

Distance (m)	Component	Dimension (mm)	Description / comments
27.0	Slit	1 × 1	White beam slit
28.0	Mirror (M1)	10 × 400	Vertical, downward reflecting, flat mirror
29.0	Mirror (M2)	10 × 400	Vertically focusing mirror, upward reflecting
31.7	DCM	10 × 200	Double-crystal monochromator
32.7	DMM	10 × 200	Double-multilayer monochromator
35.0	Mirror (M3)	10 × 200	Horizontally focusing, outward reflecting
55.0	BDA-V	NA	Vertical beam-defining aperture
64.0	BDA-H	NA	Horizontal beam-defining aperture
220.0	NF-KB	10 × 400 (KB-V)	Nanofocusing KB mirror
		10 × 122 (KB-H)	
220.3	Window		Beryllium window
220.4	Sample		Sample position

**Table 6:** Summary of the *ab initio* calculation results of the powers on BDAs at the ISN beamline

Position	Monochromator	Horizontal beam size, **Σ**_H_ (µm)	Vertical beam size, **Σ**_V_ (µm)	Peak power density (W mm^−2^)
BDA-V	DCM	42.3	6.3	142
	DMM	62.4	7.0	4790
BDA-H	DCM	14.1	31.1	85
	DMM	14.5	59.4	2470

**Table 7:** Summary of the *ab initio* calculation results of the powers on the first elements of both DCM and DMM monochromators at the ISN beamline

	Absorbed power from harmonic (W)			
Monochromator	1	2	3	4
	(5.67 keV)	(11.3 keV)	(17.0 keV)	(22.7 keV)
DCM	75.2	16.9	68.9	41.8
DMM	72.3	17.1	49.3	41.9

**Table 8:** Summary of analytical results of the power calculation on BDAs at the ISN beamline

Position	Monochromator	Horizontal beam size, **Σ**_H_ (µm)	Vertical beam size, **Σ**_V_ (µm)	Peak power density (W mm^−2^)
BDA-V	DCM	44.5	4.9	177
	DMM			9377
BDA-H	DCM	12.7	31.8	95
	DMM			5065

**Table 9:** Characteristics of undulator U21 at APS-U

Period (mm)	Number of periods	*K*	*E*_1st_ (keV)	Electron beam					
				*E* (GeV)	*I* (mA)	σ_*x*_ (µm)	σ_*y*_ (µm)	σ_*x*_^′^ (µrad)	σ_*y*_^′^ (µrad)
21	220	1.121	10	6	200	14.8	3.7	2.8	1.5
		0.980	11						
		0.845	12						

**Table 10:** List of simulated elements of the XPCS beamline

Distance (m)	Component	Dimension (mm)	Description / comments
25.6	Mask	2 × 1	Front-end mask
28.0	Mirror (M1)	10 × 490	Horizontal, outward reflecting, flat mirror
30.6	Mirror (M2)	10 × 490	Horizontal, inward reflecting, flat mirror
51.5	CRL	NA	Transfocator, Be parabolic lenses, 2D focusing
53.0	Shutter	NA	Photon shutter (PS)

**Table 11:** Transfocator configurations to focus on the PS, and calculated focus position and beam

Energy (keV)	Transfocator configurations *N* × radius (µm)	Focal position from PS (mm)
10	10 **×** 100 + 2 **×** 500	+2.8
11	12 **×** 100 + 1 **×** 200 + 1 **×** 1000	+4.7
12	14 **×** 100 + 2 **×** 200 + 1 **×** 1000	-1.1

**Table 12:** CRL specifications

Apex radius (µm)	Lens diameter (mm)	Lens thickness (µm)	Piling thickness (mm)
100	0.632	30	2.5
200	0.894		
500	1.414		
1000	2.000		

**Table 13:** Summary of the *ab initio* calculation results for the pink beam power focused by the transfocator on the photon shutter at the XPCS beamline

First harmonic *E*_1st_ (keV)	Power at PS in a 20 µm × 20 µm area (W)	Peak power density (W mm^−2^)	FWHM at PS (µm)
10	10.1	5.6 × 10^6^	1.5 × 0.8
11	11.0	5.9 × 10^6^	1.6 × 0.8
12	9.1	3.6 × 10^6^	1.7 × 0.8

**Table 14:** Summary of the analytical calculation results for the pink beam power focused by the transfocator on the photon shutter at the XPCS beamline

First harmonic *E*_1st_ (keV)	Power at PS in a 20 µm × 20 µm area (W)	Peak power density (W mm^−2^)	FWHM at PS (µm)
10	8.7	2.4 × 10^6^	1.4 × 0.7
11	14.7	4.0 × 10^6^	1.4 × 0.7
12	12.7	3.5 × 10^6^	1.4 × 0.7
